# Photosharing websites may improve Hemiptera biodiversity knowledge and conservation

**DOI:** 10.3897/zookeys.319.4342

**Published:** 2013-07-30

**Authors:** Marta Goula, José-Manuel Sesma, Luis Vivas

**Affiliations:** 1 Universitat de Barcelona, Departament de Biologia Animal, Facultat de Biologia, Avda Diagonal 643, 08028 Barcelona, Spain; 2 Invertebrate Gallery at Biodiversidad Virtual Website. Barcelona, Spain; 3 Invertebrate Gallery at Biodiversidad Virtual Website. Valencia, Spain

**Keywords:** Macrophotography, entomology, Hemiptera, new technologies, Public Participation in Scientific Research, photosharing websites

## Abstract

Internet photosharing websites is a very recent and powerful tool for the study of biodiversity, and a meeting point of general public fond of nature and professional naturalists. The article discusses when an uploaded picture is scientifically valuable, and the benefits of structured hosting websites for the most fruitful information retrieval. Examples are given of faunistic, biological, ecological and conservation results concerning Hemiptera provided by information download from photosharing websites.

## Introduction

Illustration in science has always been an indispensible tool to make scientific communication more efficient. This general statement is particularly true regarding natural sciences, as for example geology, botany or zoology. Until the invention of photography (or its eve) in the 1810’s by Niepce, the only chance for representing any subject were designs made by hand, either by the naturalist himself if picture-skilful, or by a designer collaborating with the naturalist to make scientifically valuable designs by highlighting the distinctive traits (Alamany 1997).

Photography was used by naturalist scientists very soon after being invented. From then onwards, and in a relatively short time, to take good pictures either in the field or in the laboratory has become progressively easier. The turning point was the advent of digital photography in the 1970’s, which has made everyone of us a potential photographer on any subject, including nature (Marshall 2008). Accessibility of tools needed for digital photography, together with the ease of use and accessible prices of digital devices are driving factors in the increasing amount of nature pictures shot daily all over the world.

Generalized access to digital macro photography and to internet has greatly increased the uploading of insect images to internet photo sharing websites, founding a democratic revolution in the study of biodiversity (Marshall 2008). Wilson (2004) highlighted the powerful effect of combining both technologies. Google Search “Images Hemiptera” shows 614.000 results, “Images Heteroptera” 226.000 results, “Images True Bugs” 44.400.000 results, “Images Homoptera” 137.000 results, and “Images bugs” 256.000.000 results [accessed 14 November 2012]. A certain percentage of the images retrieved does not fit the hemipterist’s target, as for instance erroneously identified pictures. However, the great bulk represents a huge image data base that hemipterists can no longer ignore, and hopefully in many cases may valuably help to the hemipterists’ work in some way.

The aim of this work is to highlight how Heteropterists’ research may be enhanced by implementing information provided by scientifically valuable uploaded pictures, in a fruitful collaboration between professional and amateur entomologists as an exercise of general public participated science.

## When a Hemiptera uploaded photo is scientifically valuable?

Internet hosts a very high number of websites whose main focus is insect macro photography. A Google search under “Hemiptera photo gallery” showed approximately 41.600 results [accessed 14 November 2012].

Uploaded macro photographs may be scientifically valuable when they provides (a) shooting date; (b) shooting site data, including georeferenced data in current units as UTM or Latitude/Longitude; and (c) features needed to identify the specimen to species level. It is worth to note that date and site shot requirements parallel the field data stated in classical insect collection labels (Marshall 2008). Many uploaded insect pictures do fit the first two requirements i.e. shooting data. Unfortunately, high-quality beautiful insects shots are too often uploaded for the sake of aesthetics and not entomology, so identification of specimen is not possible. A great improvement in this matter could be achieved by a jointed collaboration of insect amateur photographers with professional entomologists, who could give advice on external characters taxonomically valuable for each insect group. Extra information contributing to the picture interest would be altitude, habitat, host plant, or other details which inform on the species biology. Reliability of data associated to uploaded pictures relies on photographer’s ethics, as it has been also the case for centuries with author’s ethics on published data. Carefully considered, uploaded pictures allow taking a glimpse of the specimen!

## What are isolated uploaded Hemiptera images good for?

An identifiable Hemiptera image deprived of shooting date and site may still have a value for the professional hemipterists in certain circumstances. For example, images uploaded in the popular photosharing website BugGuide (http://bugguide.net/node/view/15740), may illustrate a scientific article, provided the contributor’s license terms are respected. Identification reliability may be confirmed by the own borrower hemipterist taxonomic knowledge.

Also, pictures without shooting data are the rule in online visual identification guides. Generally speaking, websites hosting visual guides are organized according to a taxonomic system, either explicit in the website, as for example in the “Heteroptera of Slovenia” (http://www2.pms-lj.si/heteroptera/), or implicit in the taxonomic presentation order of uploaded photos. Examples of the latter are “British Bugs online identification guide of UK” (http://www.britishbugs.org.uk/links.html), or the corresponding websites for German bugs (Die Familien der Wanzen http://www.golddistel.de/wanzen/index.htm), Flanders bugs (Wantsen (Heteroptera) uit onze region http://users.telenet.be/roeland.libeer1/wantsen%20web/wantsen.htm) or Austrian Auchenorrhyncha (Auchenorrhyncha http://gallery.kunzweb.net/main.php?g2_itemId=258), among others. More sophisticated taxonomic digital tools are photographic keys (e.g. Umarán and Muñoz 2012 among many other examples), an application of new technologies that has inspired the specialized Canadian Journal of Arthropod Identification (http://www.biology.ualberta.ca/bsc/ejournal/ejournal.html). Last, Hemiptera pictures not labeled with date and site may be a very good complement to specialized taxonomic websites, for instance the Plant Bug Inventory website (http://research.amnh.org/pbi/), or the broader-scoped Encyclopedia of Life website (http://www.eol.org) in which image databases are intended to be progressively built. The driving force of uploading macro photos in visual guides or specialized Hemiptera websites is to illustrate, improve or facilitate knowledge of their taxonomy. Thus, picture uploading is limited by the geographic area scoped by the website (i.e. Heteroptera from the Iberian Peninsula http://www.flickr.com/groups/iberianbugs/). Also, to guarantee a reliable species identification, only Hemiptera experts can contribute to the website. Reliable visual guides and specialized websites may help professional hemipterists when studying specimens out of their usual geographic area of study.

The use of isolated uploaded pictures becomes much more fruitful when they at least provide information on the shooting site and date. The use of these pictures ranges from species conservation to faunistics or biology.

Conservation photography, a growing developing discipline stemmed from nature photography, creates pictures to serve the purpose of conserving nature (Mittermeier 2006). The increasing importance of conservation photography is highlighted by the foundation of the International League of Conservation Photographers (iLCP’s) (http://www.ilcp.com/) and the creation of the biennial International Conservation Photography Awards (ICP Awards) (http://www.icpawards.com/index.php). A high-quality Hemiptera picture portraying a red-listed species contributes to its conservation when the associated data enlarges its distribution area, or confirms its existence after a long period of time without being found. For instance, *Parahypsitylus nevadensis* E. Wagner, 1957 (Miridae: Orthotylinae) is an Iberian endemic species which deserves the status of “vulnerable” in the Spanish Red List. Uploaded pictures (Jiménez 2010 http://www.biodiversidadvirtual.org/insectarium/Parahypsitylus-nevadensis-img144923.html) report the species more than 50 years after being described (Baena 2011).

**Figure 1.**
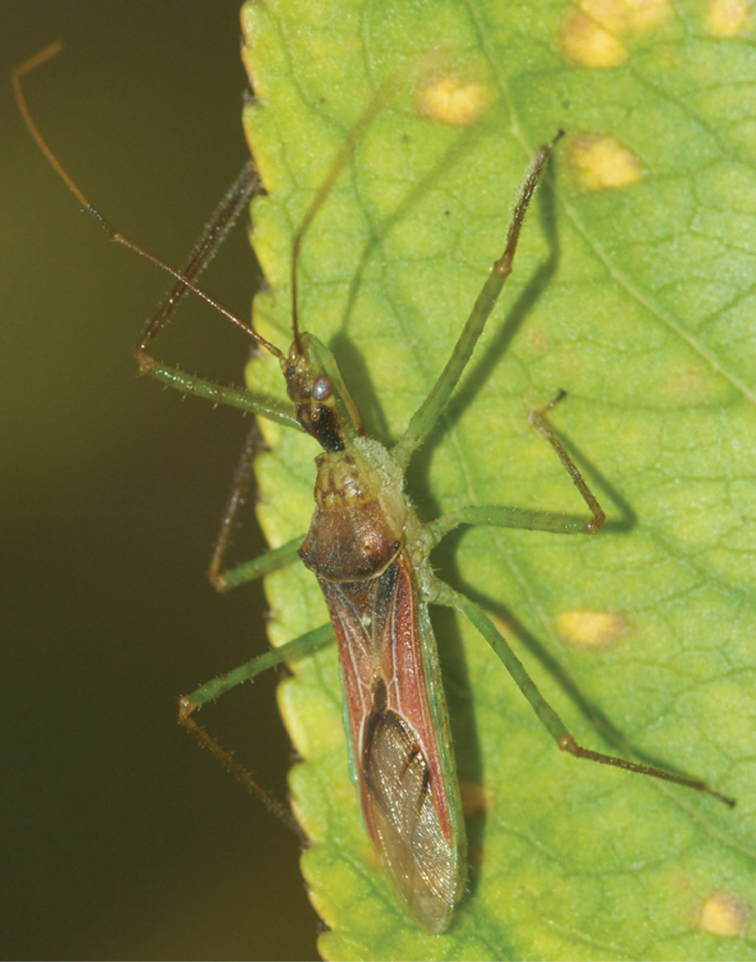
*Zelus renardii* Kolenati, 1856 (Reduviidae, Harpactorinae). Photo K. Kamppeter. Determination L. Vivas.

Faunistics may benefit from uploaded, fully labelled photographies. A great effort is being made to catalogue all biota. Nowadays technology, with which creation of digital insect collections is much facilitated, may heavily contribute to this purpose (Wilson 2004). It is estimated that the remaining 90% unknown species will be discovered within the time of a human generation (25 years). In a more limited scope, partial Hemiptera catalogues may be enlarged, or doubtful occurring species confirmed, by contributed photographies. *Zelus renardii* Kolenati, 1856 (Reduviidae, Harpactorinae) (Fig. 1) is an American species aloctonous to the Iberian fauna. In 2010 the species was found in Europe for first time (Davranoglou 2011). The species was reported in the Iberian Peninsula by uploaded pictures (Vivas 2012a). The presence of *Heegeria tangirica* (Saunders, 1877), an eremic Alydidae scarcely recorded in the Iberian Peninsula, is fully confirmed in this geographic area by uploaded pictures (Burger 2011 http://www.biodiversidadvirtual.org/insectarium/Heegeria-tangirica-img400707.html).

Biology of a species may be described or better profiled with uploaded pictures documenting host plant or habitat, or labeling altitude of shot. Dated pictures may enlarge the known species period of activity or increase the knowledge of the species phenology.

Last and beyond the purpose of macrophotographies currently available internet photosharing websites, is the uploading of multiple-view high-resolution images to produce e-types. The profit to all taxonomists is out of doubt, as accessing available e-types is a quicker and afordable procedure than visiting museums or asking for type specimens loans (Wilson 2004). Harvard University launched in 2006 the “E-Type initiative”, a 25 years project aiming at developing “Web-accessible electronic cataloguing and imaging of primary type specimens that are available for use by taxonomists and others in the research community” (Harvard University http://insects.oeb.harvard.edu/etypes/index.htm). Presently, 33 Hemiptera type-specimens may be downloaded [accessed 15 November 2012].

## Website structure helps to enhance the scientific value of uploaded pictures

The examples presented in the previous section refer to Hemiptera macro photos hosted in simply structured websites, either with or without general public contributions. In these websites, photographs may be very well documented, but they are unlinked from each other, so that retrievable information, although valuable, is very limited.

Much more flexible and fruitful information may be retrieved when the hosting website links pictures and associated data to a data base file, i.e. an excel file. The turning point is how to ensure that contributing photographers will include required associated data in the uploading process. The description given below on photosharing procedures is taken from the Spanish internet photosharing website “Biodiversidad Virtual” (http://www.biodiversidadvirtual.org/).

**Figure 2.**
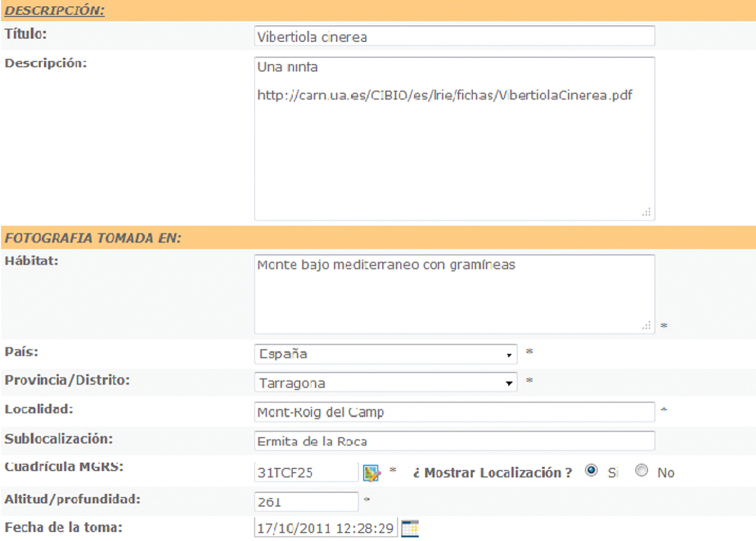
Fields to be filled when uploading pictures to Biodiversidad Virtual internet photosharing website. An asterisk (*) states for obligatory fields.

As the first step, when uploading a picture the author is constrained to fill a list of obligatory fields, the shortest list being (Fig. 2) shooting date and locality, including country, district or province and georeference data through the selection of the locality on a digital cartography. Also very valuable are habitat description and altitude.

Once in the photosharing website system, the picture is confidently identified. Tentative identification may be proposed by the author, or by the website administrators. Checking by an expert is always advisable and compulsory when identification is not so straightforward. Thus, part of the website administrators’ job is to organize a network of experts who may attend consultations.

**Figure 3.**
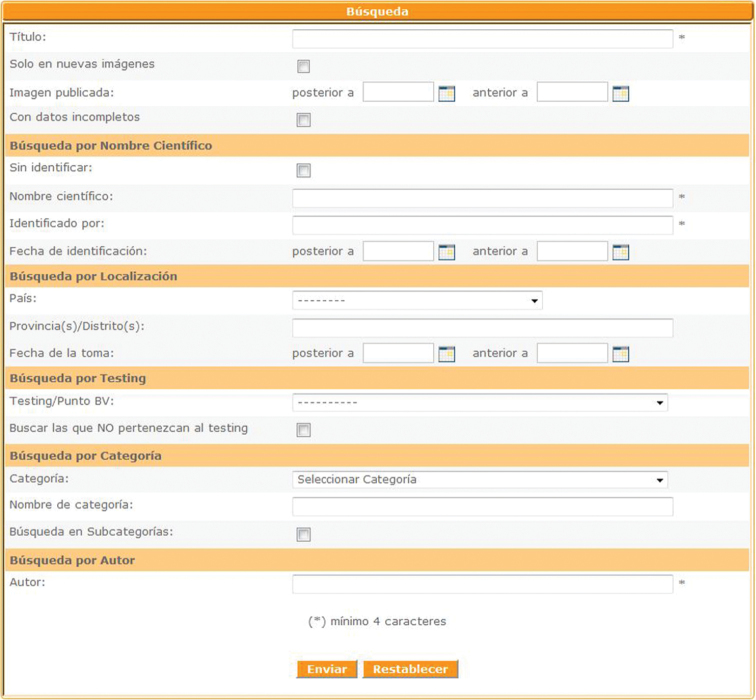
Windows interface implemented in Biodiversidad Virtual internet photosharing website to retrieve information from database set.

Filled data and species identification are automatically downloaded to an excel file, so that data linked to each uploaded photograph will contribute to website database. A friendly windows interface allows consulting photo sharing website database according to one or more filled fields (Fig. 3), and a large amount of new information may be generated, including all the cases commented before in relationship with isolated uploaded pictures. For the sake of simplification, only a few examples, not overlapping those previously stated, are given below. Examples will deal with conservation, faunistics, biology and ecology. Describing completely the retrieval power of a database-linked photosharing website is beyond the scope of this article.

### Conservation

**Figure 4.**
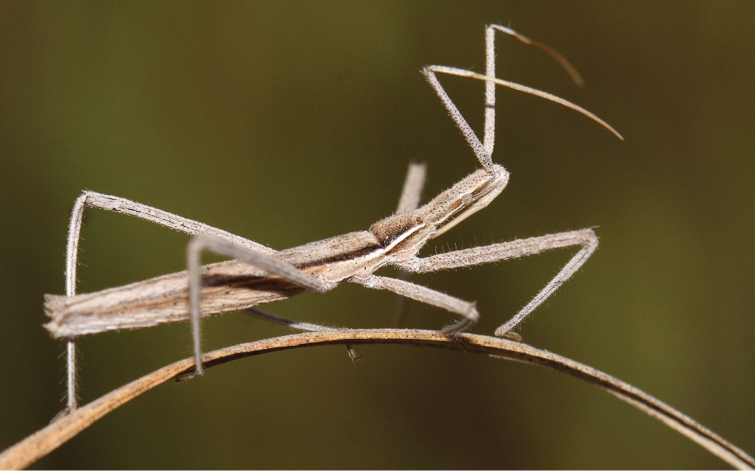
*Vibertiola cinerea* Horváth, 1909 (Reduviidae, Harpactorinae). Photo JM Sesma. Determination L. Vivas.

*Vibertiola cinerea* Horváth, 1909 (Reduviidae, Harpactorinae) (Fig. 4) is a Mediterranean species extending to the Sinai and Yemen. *Vibertiola cinerea* is presentd as Vulnerable (D2) in the Spanish Invertebrate Red List (Ribes Español et al. 2011). Two out of the seven localities stated are known only by photographies uploaded in the photosharing website Biodiversidad Virtual, enlarging the already known Iberian distribution area of *Vibertiola cinerea* ca. 150km southwards.

### Faunistics

**Figures 5–7.**
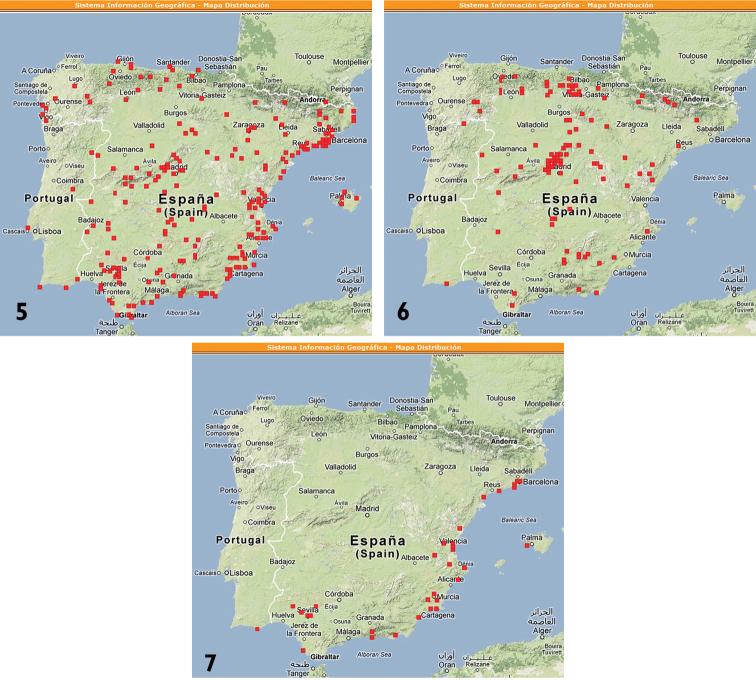
**5** *Spilostethus pandurus* (Scopoli, 1763) (Lygaeidae, Lygaeinae) **6** *Spilostethus saxatilis* (Scopoli, 1763) (Lygaeidae, Lygaeinae) **7** *Spilostethus furcula* (Herrich-Schaeffer, 1850) (Lygaeidae, Lygaeinae) citations according to retrieved information from 399 pictures hosted at Biodiversidad Virtual internet photosharing website.

*Spilostethus* is a bigsized Lygaeinae genus with only three species belonging to the Iberian fauna. *Spilostethus pandurus* (Scopoli, 1763) and *Spilostethus saxatilis* (Scopoli, 1763) are very commonly found, but *Spilostethus furcula* (Herrich-Schaeffer, 1850) is scarcely observed along the Iberian Mediterranean climate area. All three species show a bright black and red color pattern, which may mislead a non-expert observer. Size and color make them a frequent target for macro photography, while *Spilostethus* species are often neglected in field trip collections as they are considered frequent and banal. Vivas (2012b) compiled information from georreferenced pictures in “Biodiversidad Virtual”, and charted a high number of localities for all three Iberian *Spilostethus* species (Figs 5–7).

### Ecology

*Spilostethus furcula* (Lygaeidae) is an afrotropical species, extending to the Maghreb and the Iberian Mediterranean coast. Northern Iberian localities, as documented in the shooting data from uploaded pictures (Fig. 7), may highlight how living conditions for this species occur now in areas previously hostile to it, thus perhaps illustrating an effect of global warming (Goula and Mata 2011).

**Figure 8.**
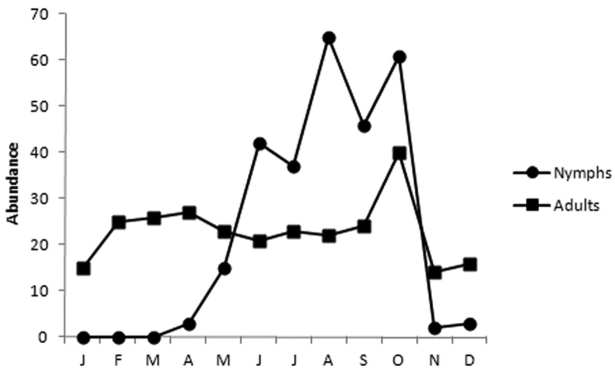
Phenology of *Nezara viridula* (Linnaeus, 1758) between December 2008 and November 2012, according to data from 550 pictures hosted in Biodiversidad Virtual internet photsharing website.

### Biology

Due to its big size, abundance and frequency, *Nezara viridula* (Linnaeus, 1758) is a largely portrayed Pentatominae. Except for the very young instars, *Nezara viridula* nymphs may be identified to species level thanks to their distinctive color pattern. 550 pictures confidently belonging to *Nezara viridula* were uploaded to Biodiversidad Virtual from December 2008 to November 2012. Nymphs and adults were equitably represented in this pool. Summarizing shooting dates results in a phenology graph (Fig. 8), which shows that adults may overwinter, and nymphs are observed from April to October, with maximum nymphs numbers in summer.

## What are the limits of photosharing website databases?

Pictures shot and uploaded to a photosharing website do not arise from a previously planned sampling. Most probably, the contents on a photosharing website are biased towards the most colorful, bigsized, abundant or frequent species. Moreover, the distribution of shoots is uneven within any given territory. For example, natural protected areas, due to its potentially more interesting and diverse biota, may differentially attract nature photographers. Also, accessibility by private or public transportation means may favor certain areas over others, or the generally uneven distribution of human population may result in more shoots in accessible or crowded areas *vs*. less shoots in the isolated or uncrowded areas. Holiday periods will contribute to uneven shooting activity along the year, as also will cold and warm months. Compilation of all published data on any ubiquitous Hemiptera species concerning Catalan territory shows that field results are also spatially unvently distributed (Biodiversity data bank of Catalonia http://biodiver.bio.ub.es/biocat/index.jsp#pas18). Last but not least, many species will never be identifiable in a picture, as examination of genitalia is the only reliable method to verify species identity. With these handicaps in mind, some of them severely affecting the quantification of the retrieved information, internet photosharing websites are still a useful and valuable complementary source of information for the professional hemipterist.

## Phtosharing websites, science and society

An increasing number of non-biologist amateurs are approaching nature in general, and insects in particular, by the practice of macrophotography. In fact, photo shooting and sharing is an accessible way to enjoy nature. When pictures are hosted in a website appropriately designed as previously described, the pleasure of photosharing increases with the pleasure of contributing scientifically valuable information. In fact photo sampling may bring together the general public, fond of nature and photography, to the scientific world, in an exercise close to Public Participation in Scientific Research (PPSR), lacking in this case an intentional design and a previous training (Shirk et al 2012). However, administrators of internet photosharing website may launch intentional designed projects, as for example occurred when the administritators of Biodiversidad Virtual website called the community to perform photosampling specifically on Hemiptera specimens during June 2011. In this case, 2340 pictures were uploaded, belonging to 170 species, 17 of them new for the website (Angulo 2011). On the other hand, scientists may profit froom photosharing websites by accessing extra information at very low effort and cost, and at disposal at the shortest period of time.

## Conclusions

Scientific information has traditionally being retrieved from specialized books and journals. In the case of entomology, the publication of field sampling results has been the cornerstone of biodiversity datasets. Availability of modern digital photograph technologies, together with worldwide access to internet is profoundly modifying the study of biodiversity. Hemiptera is a good target group to be approached through the combination of these news technologies in reason of their frequency, and some results may be already retrieved from uploaded photographic data.

Full retrievable information power from internet photosharing websites is still to come. Biases and pitfalls due to unplanned photosampling underpinning uploaded photographies will always handicap websites. Website administrators may contribute to minimize those undesirable imperfections by encouraging specific photosampling addressed to areas, groups or periods of time underrepresented in the website database. However, only website database exploitation coherent with those intrinsic limitations may give scientifically fruitful and valuable results.

Internet photosharing websites are a pleasant, accessible and encouraging tool to implement Public Participated Scientific Research in relationship to Hemiptera biodiversity. Much work is needed to catalogue, document and portray the ca. 40.000 estimated bug species living on earth. The help of thousands of volunteer macro photographers uploading their valuable high-quality pictures should not be discarded.
